# Identification of biomarkers between coronary artery disease and non-alcoholic steatohepatitis: a combination of bioinformatics and machine learning

**DOI:** 10.3389/fgene.2025.1573621

**Published:** 2025-07-17

**Authors:** Yihong Lin, Jingmei Song, Xiaohong Li

**Affiliations:** ^1^The Second School of Clinical Medicine, Zhejiang Chinese Medical University, Hangzhou, China; ^2^School of Basic Medicine, Zhejiang Chinese Medical University, Hangzhou, China; ^3^School of Pharmaceutical Sciences, Zhejiang Chinese Medical University, Hangzhou, China

**Keywords:** coronary artery disease, non-alcoholic steatohepatitis, machine learning, WGCNA, bioinformatics

## Abstract

**Background:**

Non-alcoholic steatohepatitis (NASH) commonly complicates coronary artery disease (CAD), yet the interaction mechanism remains unclear. Our research seeks to investigate the common mechanisms and key signature genes between CAD and NASH.

**Methods:**

RNA sequence information for CAD and NASH was screened from the GEO database. Weighted gene co-expression network analysis (WGCNA) and differentially expressed gene analysis identified key genes, followed by functional enrichment analysis of these shared genes. Three machine learning methods—LASSO, random forest, and SVM-RFE—were used to identify signature genes. Gene set enrichment analysis (GSEA) was then performed to explore potential mechanisms associated with the signature genes. In addition, single-sample gene set enrichment analysis (ssGSEA) evaluated immune infiltration in CAD and NASH and its correlation with the signature genes.

**Results:**

WGCNA has revealed two key modules for CAD and NASH. The intersection of the CAD modules and their differential genes narrowed the key genes down to 2,808 shared genes. Finally, 44 shared genes were selected for both CAD and NASH. Kyoto Encyclopedia of Genes and Genomes analysis showed that these genes were primarily enriched in insulin resistance and inflammation pathways. Machine learning identified the signature genes BATF3, SOCS2, and GPER, all with ROC values above 0.7, validated in external datasets. GSEA revealed that these genes act through common mechanisms in CAD and NASH, regulating metabolic, inflammatory, and cardiovascular pathways. In addition, ssGSEA suggested their involvement in immune cell infiltration.

**Conclusion:**

BATF3, SOCS2, and GPER have emerged as promising gene candidates that may serve as biomarkers or potential therapeutic targets for CAD combined with NASH, linked to the regulation of metabolic, inflammatory, and cardiovascular pathways. We also identified insulin resistance and inflammation pathways as common mechanisms underlying both diseases.

## 1 Introduction

Coronary artery disease (CAD) is a significant type of cardiovascular disease (CVD) and one of the leading causes of global mortality and the loss of disability-adjusted life years (Ralapanawa and Sivakanesan, 2021). There are several risk factors that can lead to CAD. These include family history, smoking, alcohol consumption, diabetes, age, obesity, and hypertension (Madhavan et al., 2018). The development of atherosclerotic plaques is the main pathological feature of CAD. This process involves the continuous deposition of excess cholesterol and cholesterol esters in the arterial intima, resulting in the proliferation of connective tissue, thickening and hardening of the arterial wall, and subsequent connective tissue necrosis (Chiu et al., 2018).

Non-alcoholic fatty liver disease (NAFLD) has emerged as the most common chronic liver condition in adults globally, affecting roughly 25% of the population (Powell et al., 2021). NAFLD represents a significant public health issue, contributing greatly to liver-related morbidity and mortality. In addition, it has become the most rapidly increasing cause of hepatocellular carcinoma. Non-alcoholic steatohepatitis (NASH) is a more severe form of NAFLD. It is characterized by the development of hepatocellular ballooning, lobular inflammatory infiltrates, and fibrosis developing atop simple hepatic steatosis (Książek et al., 2024). Current studies have found that the incidence of cirrhosis in NASH patients within 10–15 years is ten times higher than that in patients with simple steatosis, reaching 15%–25%. Moreover, the five-year and ten-year survival rates for patients with NASH are 67% and 59%, respectively (Wang et al., 2014; Fan et al., 2019). Therefore, NASH is the primary target for clinical interventions aimed at preventing the progression of NAFLD and represents a critical stage in the progression of liver disease in NAFLD patients. Patients with NAFLD exhibit cardiometabolic risks comparable to those observed in individuals with CAD, including inflammation, dyslipidemia, and endothelial dysfunction. The presence of NAFLD may serve as a considerable risk factor for the advancement of CAD (Zhang et al., 2020; Zhang and Fang, 2021). One study found that among the subtypes of NAFLD, patients with NASH pose a greater risk of dying from CVD than those with simple steatosis alone (Vanni et al., 2015).

A robust correlation exists between NASH and atherosclerotic cardiovascular disease. NASH has been demonstrated to contribute to the onset and progression of CVD. This is accomplished through inflammatory mediators released systemically (e.g., TNF-α, CRP, PCF) and oxidative stress markers (Targher et al., 2010). Nevertheless, the precise mechanisms through which NASH contributes to the development of atherosclerosis remain poorly understood. The molecular biology research on the shared mechanisms of the two diseases is limited. Genetic variants in PNPLA3, TM6SF2, and GCKR have been proposed as potential drivers of atherosclerosis in NAFLD patients, though this remains a topic of debate (Petta et al., 2013; Dongiovanni et al., 2015; Xia et al., 2016). Acute myocardial infarction constitutes an acute manifestation of CAD. A study in China found that elevated serum FSTL3 levels may increase the risk of fibrosis and acute myocardial infarction in type 2 diabetes patients with NAFLD (Duan et al., 2023). Bioinformatics studies have identified *PLCXD3, CCL19, PKD2* and *MMP9* as signature genes in atherosclerosis and NAFLD (Mo et al., 2023; Lv et al., 2024). Although NASH has received widespread attention as an important subtype of NAFLD, there is still a lack of bioinformatics studies directly focusing on the link between CAD and NASH.

This study aims to investigate the common pathogenic mechanisms of CAD and NASH by analyzing transcriptomic data sourced from the Gene Expression Omnibus (GEO) database. To this end, bioinformatics approaches and machine learning methods will be employed to explore common pathogenic pathways, signature genes, and immune infiltration profiles associated with CAD and NASH.

## 2 Materials and methods

### 2.1 GEO dataset download and process

The key terms “coronary artery disease” and “non-alcoholic steatohepatitis” were employed to search for gene expression profiles associated with CAD and NASH in the GEO database (Home - GEO - NCBI (nih.gov)). The datasets were considered eligible if they met the following criteria: high-throughput expression data, human tissue samples, and at least 10 samples per group to ensure the reliability of the weighted gene co-expression network analysis (WGCNA). Finally, the following datasets were chosen for analysis: GSE113079, GSE66360, GSE89632, and GSE135251. Of these, GSE113079 and GSE89632 were subjected to comprehensive analysis, while GSE66360 and GSE135251 were employed for validation purposes. The GSE113079 dataset comprises 93 CAD patients and 48 normal controls, while the GSE89632 dataset contains 19 NASH patients and 24 normal controls. The GSE64566 dataset (GPL6947 platform, 26 CAD cases, and 20 controls) and the GSE135251 dataset (155 NASH cases and 10 controls) were employed to validate the results further. The “limma” package in R was used to normalize the data, ensuring appropriate corrections across datasets were implemented. Probe annotations have been mapped to gene symbols by means of the annotation files provided by the respective platforms. The resulting gene expression matrices were utilized for subsequent analyses, with samples as rows and gene symbols as columns.

### 2.2 Weighted gene co-expression network analysis

The modules associated with CAD and NASH were explored using WGCNA (Langfelder and Horvath, 2008). First, hierarchical clustering was performed using the “hclust” function in R to remove outliers. The “pickSoftThreshold” function in the WGCNA package was used to search for the best soft-threshold power and adjacency according to scale-free network criteria. The adjacency matrix was converted into a topological overlap matrix, and phase differences were calculated. A hierarchical clustering dendrogram was constructed, and genes exhibiting analogous expression patterns were grouped into discrete modules. The dynamic tree-cut method was employed to construct the co-expressed gene modules, with the minimum module size set to 50. Finally, the relationship between the gene module and the disease was assessed using the gene significance and module membership values to identify the core module.

### 2.3 Identifying differentially expressed genes between CAD samples and controls

Differential expression analysis was performed on the CAD dataset to further narrow down the number of genes identified in the key CAD module obtained from WGCNA. The R package “limma” (Ritchie et al., 2015) was used to identify differentially expressed genes(DEGs) in GSE113079 using the inclusion criteria $\left|\log_{2}Fold\;Change\;\right|\geq 0.5$ and p−adjust < 0.05. Volcano plots were generated to characterize the DEGs, and the expression levels of the 50 most significantly represented genes were plotted using the heatmap. The intersection of key modules and differentially expressed genes in the CAD dataset is defined as CAD key genes. We employed the JVenn online tool to construct a Venn diagram (Philippe Bardou et al., 2014).

### 2.4 Identification and enrichment analysis of shared genes between CAD and NASH

Genes that are present in both the CAD and NASH key modules and have the same expression trend in both diseases are defined as shared genes. The visualization was carried out using Venn diagrams (Philippe Bardou et al., 2014). To investigate the potential functions of the genes shared between CAD and NASH, we performed Gene Ontology (GO) and Kyoto Encyclopedia of Genes and Genomes (KEGG) pathway analyses using the org. Hs.e.g.,.db, ggplot2, clusterProfiler, and enrichplot packages in R. GO analysis was utilized to clarify the biological processes (BP), molecular functions (MF), and cellular components (CC) involved in the overlapping genes. KEGG pathway analysis was conducted, aiding in the identification of the underlying biological mechanisms within the shared genes.

### 2.5 Machine learning and signature gene identification

The shared genes between CAD and NASH were investigated using three machine-learning methods. The goal was to find common signature genes using Least Absolute Shrinkage and Selection Operator (LASSO) Regression, random forest (RF), and support vector machine recursive feature elimination (SVM-RFE). LASSO regression is an improved form of linear regression which introduces an L1-norm penalty to the least squares objective function. This regularization shrinks some regression coefficients to zero, thereby enabling effective variable selection (Tibshirani, 1996; Ranstam and Cook, 2018). The LASSO analysis was performed using the “glmnet” package, with the penalty parameters selected through 10-fold cross-validation. RF is an ensemble learning method that enhances prediction stability and accuracy by aggregating the results of multiple decision trees (Cutler et al., 2012). In this study, gene importance scores were computed using the RF algorithm to identify candidate genes that may play a crucial role in the development and progression of disease. The “randomForest” package was used to perform the analysis. To identify the ideal number of trees for the RF algorithm executed by the “randomForest” package, the error rate was initially computed for a series of values ranging from 1 to 500 trees. Ultimately, the significance of each candidate signature gene was assessed using the “importance” function, and the top five genes were chosen according to the computed importance scores. SVM-RFE is a recursive feature elimination method based on support vector machines that iteratively trains the model and removes the least informative features to identify the most significant variables for classification (Guyon et al., 2002). The SVM-RFE algorithm was trained and evaluated using the “e1071”and “caret” packages.

The overlapping genes between the genes derived by the three machine learning algorithms were called signature genes. A Venn diagram was constructed to illustrate the overlap between the two groups. The diagnostic performance of these signature genes was evaluated using the area under the receiver operating characteristic (ROC) curve (AUC). An AUC value greater than 0.7 was considered indicative of excellent diagnostic performance, while values between 0.5 and 0.7 were considered indicative of satisfactory performance. To facilitate the visualization of gene expression in the GSE113079 and GSE89632 datasets, boxplots were generated using the R package “ggplot2”.

### 2.6 Validating key genes

Validation was conducted using the GSE66360 and GSE135251 datasets to confirm the reliability of the expression levels of these signature genes in CAD and NASH. We compared the expression levels of the signature genes between the patient and control cohorts to determine significant differences and generated boxplots for visual representation. The diagnostic performance of these signature genes was then evaluated using the AUC.

### 2.7 Gene set enrichment analysis

To analyze the role of the signature genes in these two diseases, we used gene set enrichment analysis (GSEA) with GSEA software version 4.3.2. The gene set “c2. cp.kegg_legacy.v2023.2. Hs.symbols.gmt” was taken as the reference. The genes were then separated into two groups based on the expression levels in the software, and GSEA was conducted to identify significant pathways with a p-value below 0.05.

### 2.8 Immune infiltration analysis

The variations in immune cell infiltration in peripheral blood samples from CAD and NASH patients were compared with normal peripheral blood samples using single-sample gene set enrichment analysis (ssGSEA). We calculated Spearman’s rank correlation coefficients in R for immune cells with differentially signature genes to analyze their relationship with specific genes. The CIBERSORT immunization algorithm was validated against immune cells associated with and expressing the same trend of signature genes in both diseases.

### 2.9 Statistical analysis

**FIGURE 1:**
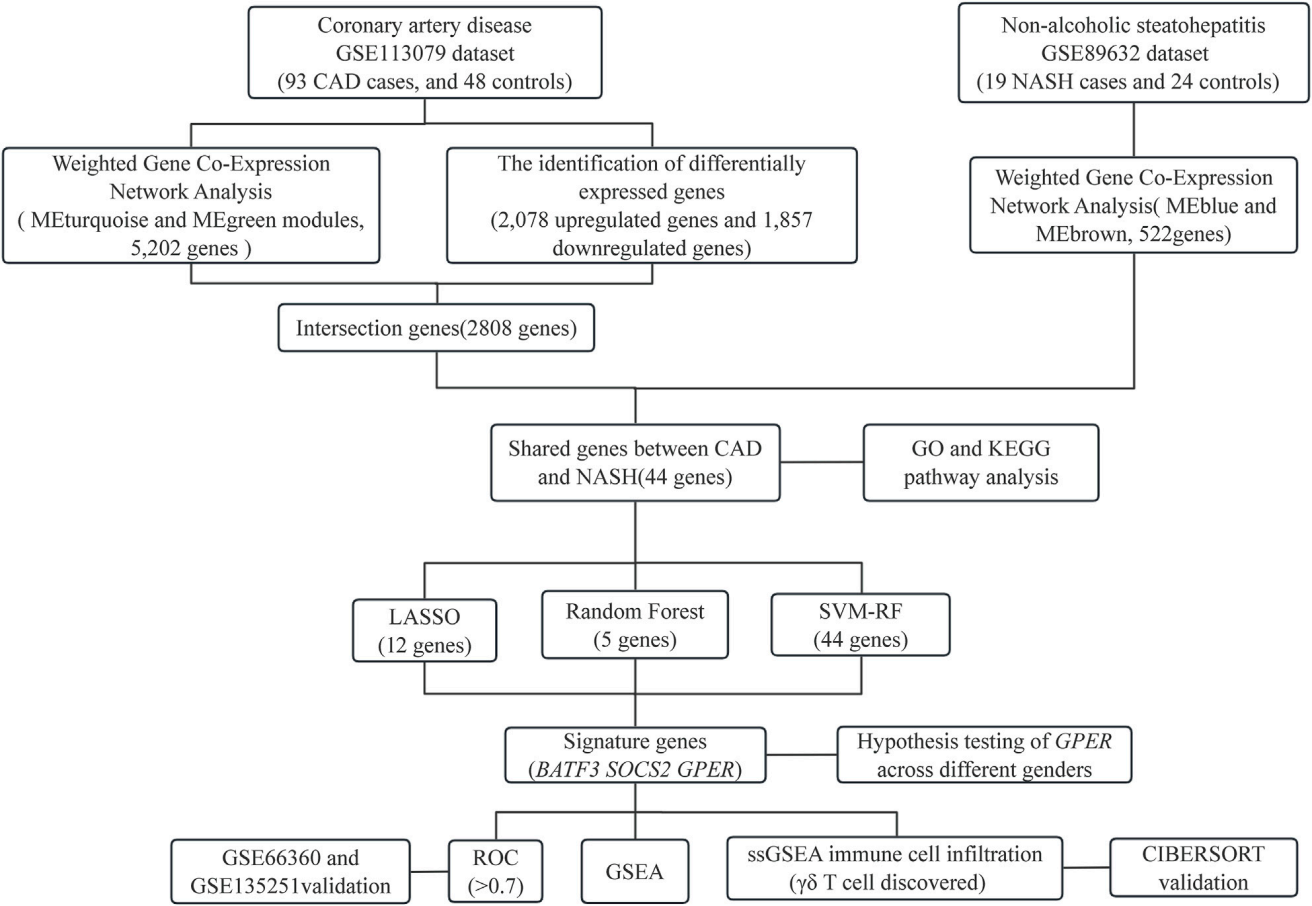
The process diagram of this research.

Statistical analyses were performed with R software (version 4.3.1). P-values below 0.05 were deemed statistically significant, and all P-values were computed as two-tailed. The flowchart in Figure 1 shows the methodology of the study.

## 3 Results

### 3.1 Identifying co-expression modules in CAD and NASH

**FIGURE 2:**
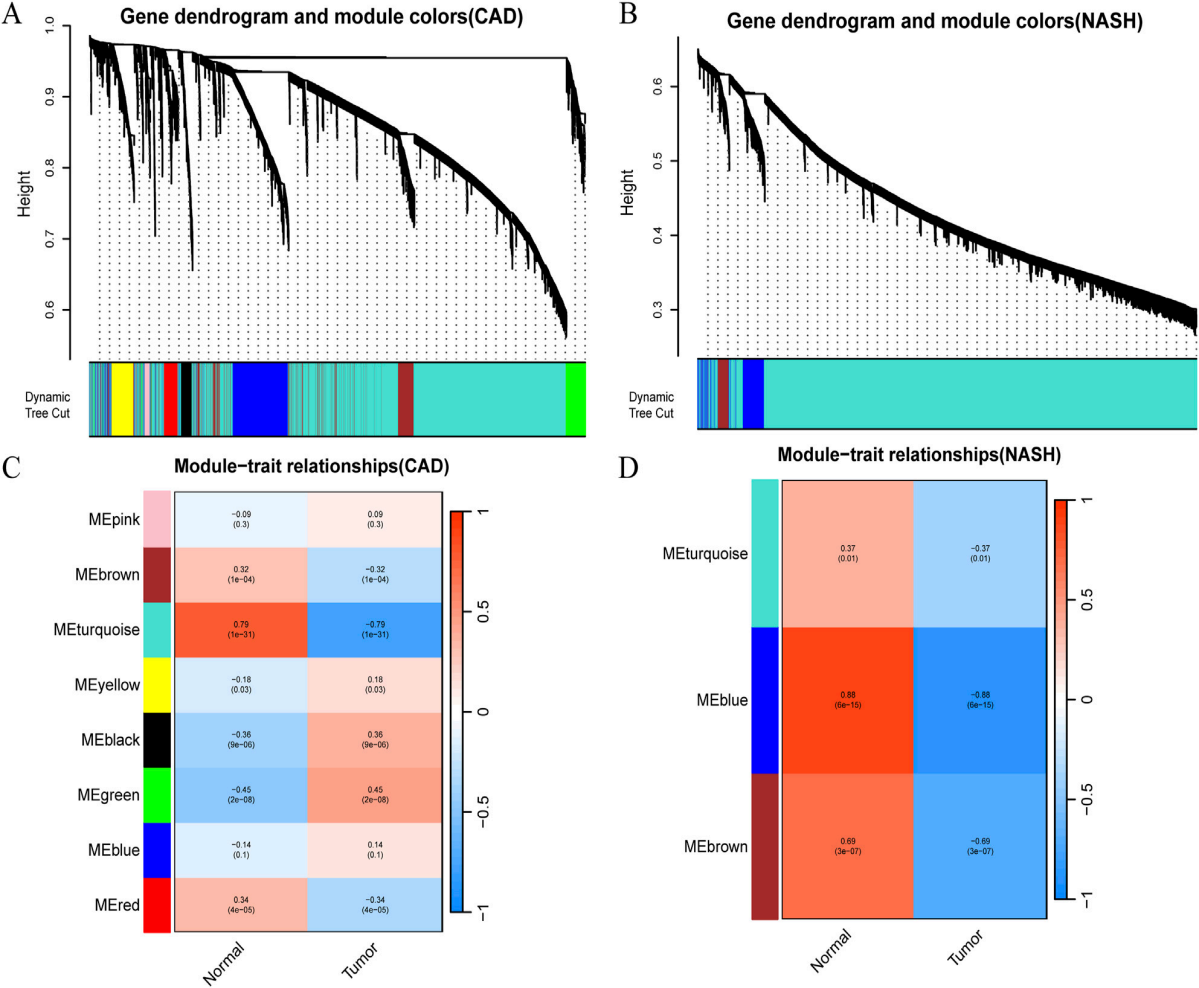
Detection of module genes using WGCNA in GSE113079 for CAD and GSE89632 for NASH. **(A)** Clustering dendrogram showing gene co-expression modules in various colors for CAD. **(B)** Clustering dendrogram for gene co-expression modules in NASH. **(C)** Heatmap of module-trait relationships in CAD, with row-column intersections indicating correlation and p-values. The x-axis represents different samples, with blue indicating healthy controls and red indicating disease group samples. The y-axis represents differentially expressed genes. Color intensity reflects the expression level of each gene across the samples. **(D)** Heatmap of module-trait relationships in NASH, with intersections showing correlation and p-values. Axes and box colors as described above.

WGCNA analysis of CAD and NASH-related genes was performed to identify key genetic modules. As a result, Spearman’s correlation coefficient was employed to create heat maps of module-trait associations. Each color represents a different module (Figures 2A,B). Figure 2C shows that eight gene modules were detected in the GSE113079 dataset. The MEturquoise module demonstrated a robust positive correlation with the disease $\left(r=0.79,p=1e-31\right)$ , whereas the MEgreen module exhibited a negative correlation $\left(r=-0.45,p=2e-08\right)$ . Accordingly, the MEturquoise and MEgreen modules were identified as CAD key modules, encompassing a total of 5,202 genes. In the GSE89632 dataset, three gene modules were identified (Figure 2D). The MEblue $\left(r=0.88,p=6e-15\right)$ and MEbrown $\left(r=0.69,p=3e-07\right)$ modules were selected as key modules for NASH, comprising 375 and 147 genes, respectively.

### 3.2 Identifying DEGs between CAD samples and controls

**FIGURE 3:**
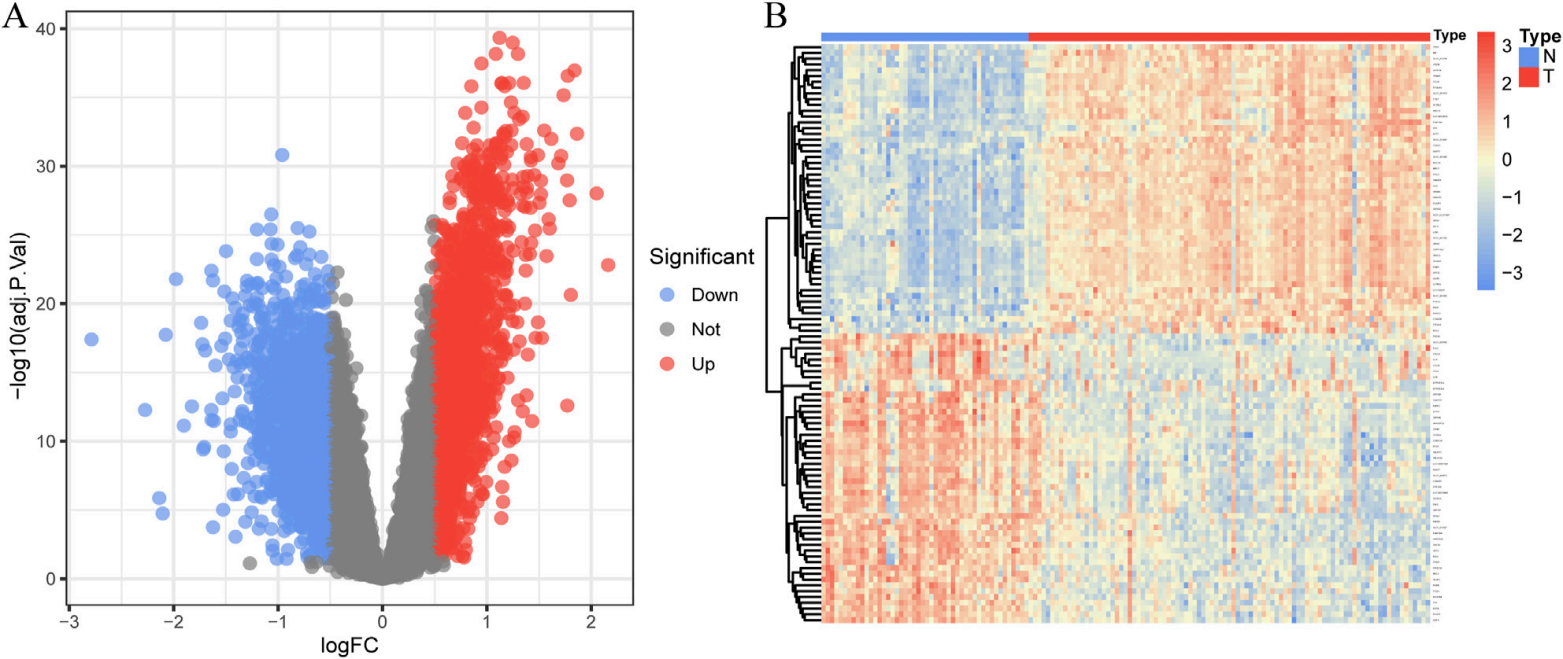
Identification of DEGs in coronary artery disease. **(A)** The volcano plot displays DEG expression between CAD and healthy groups. **(B)** Heatmap showing the top 50 upregulated and downregulated DEGs. Blue squares indicate the healthy group, and red squares indicate the disease group.

**FIGURE 4:**
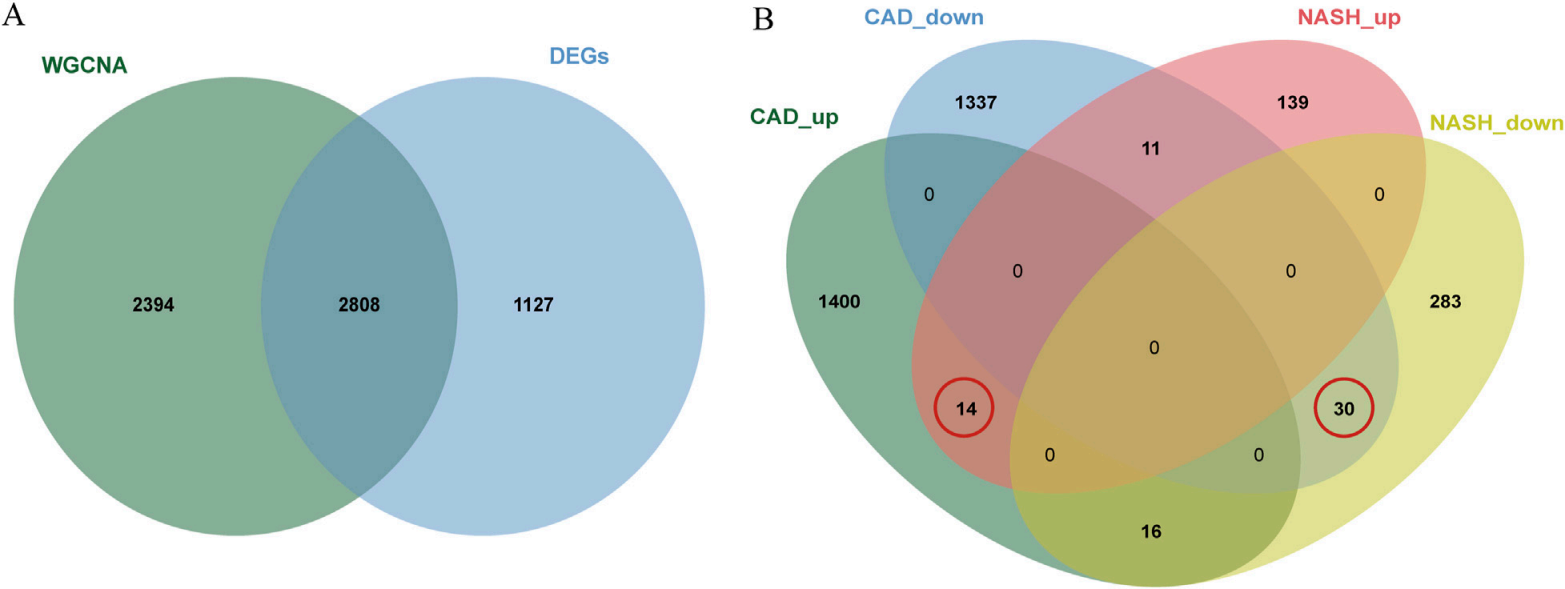
Venn diagrams for differential analysis and common gene screening. **(A)** The Venn plot illustrated the overlap between DEGs and genes identified in WGCNA. **(B)** Venn diagram of the shared genes in CAD and NASH. The gene we selected is circled in red.

In total, 3,935 genes were screened, including 2,078 upregulated genes and 1,857 downregulated genes (Figure 3A). A heatmap displaying the top 50 upregulated and DEGs between CAD samples and the healthy group is shown in Figure 3B. The intersection of DEGs in CAD and the key modules in WGCNA was taken, resulting in a total of 2,808 genes, and a Venn diagram was drawn (Figure 4A).

### 3.3 Identifying of shared gene signatures in CAD and NASH

A total of 44 genes were recognized by the overlap of the previously identified key CAD genes and the genes from the blue and brown modules of NASH (Figure 4B).

### 3.4 GO and KEGG enrichment analysis of shared genes

**FIGURE 5:**
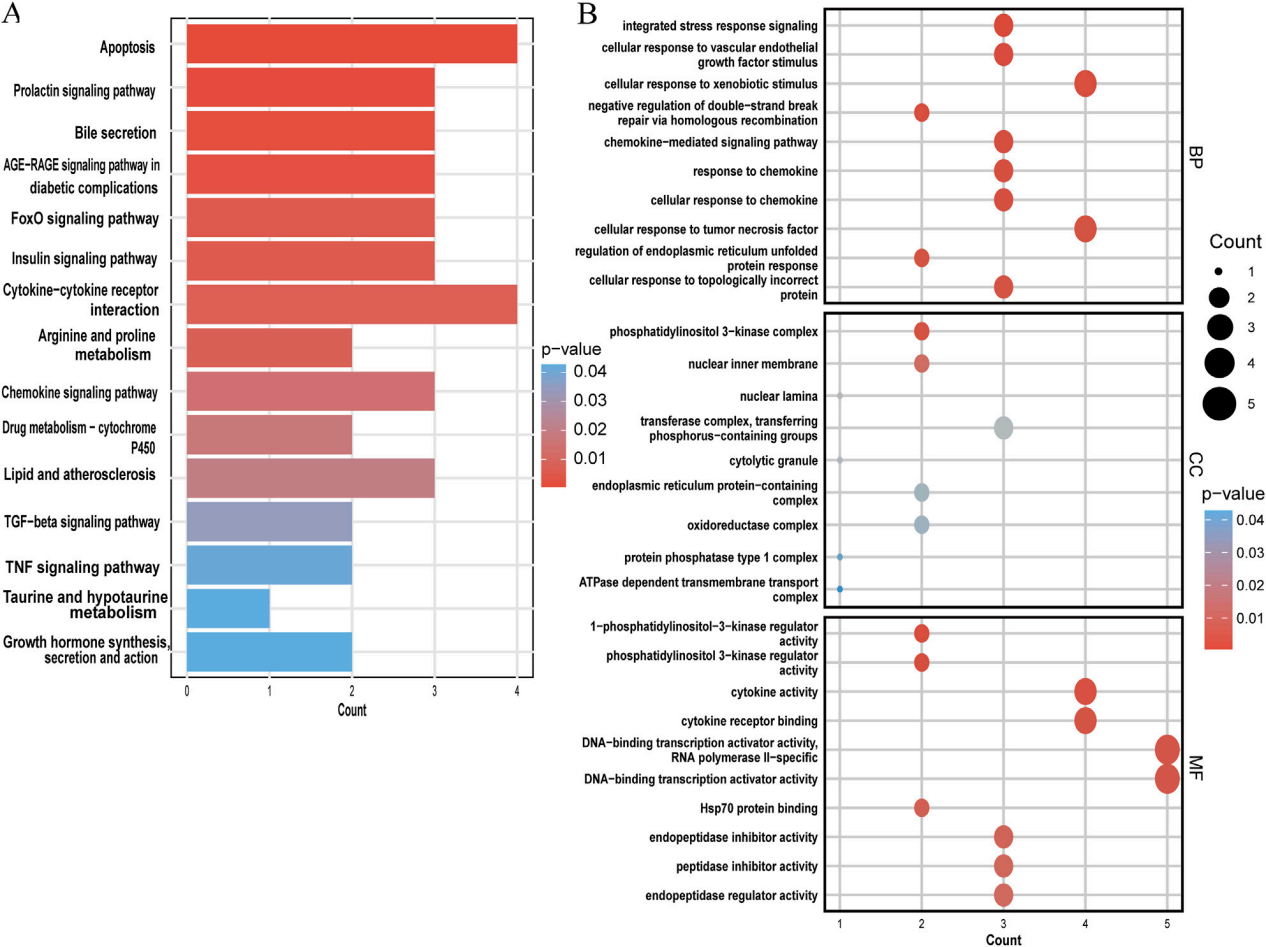
Functional enrichment analysis of shared genes. **(A)** KEGG analysis of shared genes. **(B)** The top 10 functional enrichments in each of the three GO categories. In both panels, the y-axis represents KEGG or GO enrichment pathways, and the x-axis represents the number of enriched genes. The color gradient indicates the p-value of enrichment.

The KEGG and GO analyses identified several enriched pathways relevant to the investigated diseases. In the KEGG analysis results, we displayed only the pathways related to our disease research, including apoptosis, FoxO signaling pathway, insulin signaling pathway, cytokine-cytokine receptor interaction, arginine and proline metabolism, lipid and atherosclerosis, TGF-beta signaling pathway, TNF signaling pathway, etc. and drew a bar graph (Figure 5A). Three categories make up the GO analysis: BP, CC, and MF. The BP analysis results revealed that the three most notable processes were integrated stress response signaling, cellular response to vascular endothelial growth factor stimulus, and cellular response to xenobiotic stimulus. In the CC analysis, the top three categories were identified as phosphatidylinositol 3-kinase complex, nuclear inner membrane, and nuclear lamina. In addition, 1-phosphatidylinositol 3-kinase regulator activity, cytokine activity and cytokine receptor binding were observed to have a significant role in the MF category (Figure 5B).

### 3.5 Machine learning and identification of signature genes

**FIGURE 6:**
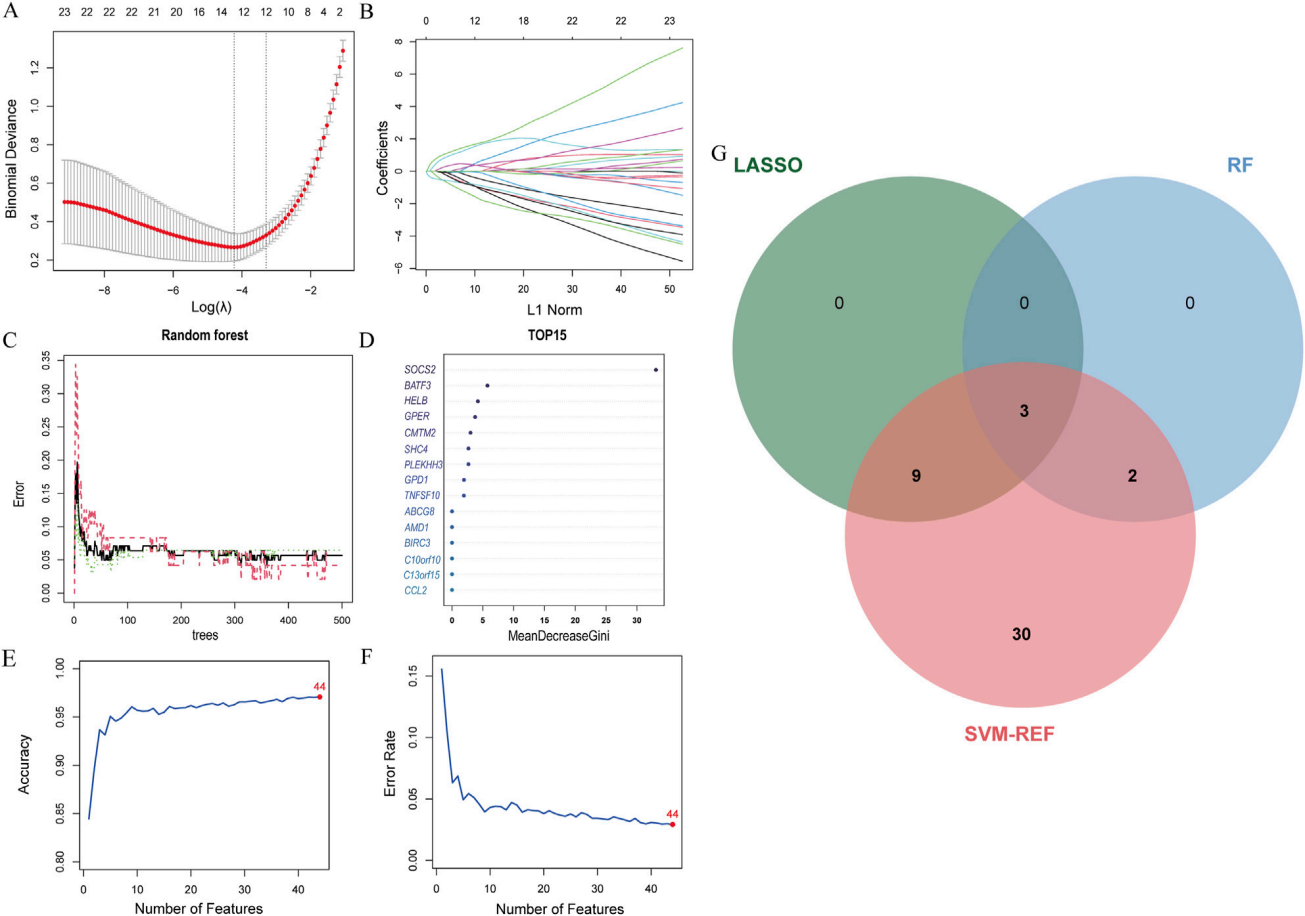
Machine learning model construction. **(A)** LASSO penalty plot with error bars for standard errors. **(B)** LASSO L1 norm path plot. **(C)** Top 15 important genes. **(D)** Random Forest error rate vs number of trees. **(E)** SVM-RFE accuracy rate curve. **(F)** SVM-RFE error rate curve. **(G)** Venn diagram of genes from the three algorithms.

**FIGURE 7:**
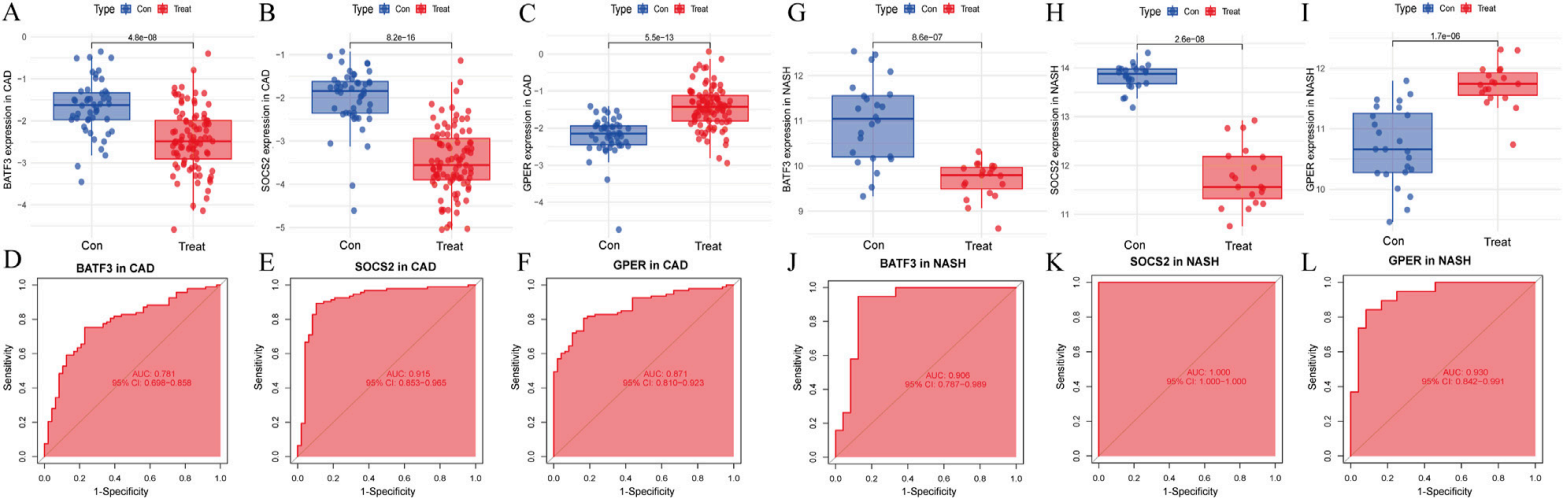
Signature genes performance in GSE113079 and GSE89632. **(A–C)** Expression levels in CAD vs healthy cohorts. The x-axis represents the sample groups, and the y-axis shows the expression level of the gene $\left(\log2\;normalized\;counts\right).$ Blue boxes represent the healthy group, and red boxes represent the disease group. **(D–F)** ROC curves showing diagnostic performance in CAD. **(G–I)** Expression levels in NASH vs healthy cohorts. Axes and box colors as described above. **(J–L)** ROC curves illustrating diagnostic performance in NASH.

The LASSO analysis revealed 12 signature genes (Figures 6A,B), whereas the RF analysis highlighted the top five most significant signature genes (Figures 6C,D). Genes selected based on LASSO are in Supplementary Table S1. The SVM-RFE analysis indicated that a model containing 44 genes demonstrated the highest accuracy (Figures 6E,F). The genes obtained from the three algorithms were ultimately found to be in common, identifying three signature genes. The final signature genes were *BATF3*, *GPER*, and *SOCS2* (Figure 6G). Both *BATF3* and *SOCS2* were lowly expressed in CAD and NASH (Figures 7A,B; Figures 7G,H). This suggests that those genes are suppressed in the disease. In both conditions, the parameter test for these two genes yielded p-values less than 0.05, indicating a statistically significant difference in expression between the normal and disease groups. In patients with CAD, the area under the AUC for the signature genes *BATF3* and *SOCS2* were 0.781 and 0.915, respectively (Figures 7D,E). In NASH, the AUC values for *BATF3* and *SOCS2* were 0.906 and 1, respectively (Figures 7J,K). Both genes exhibited AUC values exceeding 0.7, indicative of robust predictive efficacy. Conversely, *GPER* was highly expressed in both CAD and NASH (Figures 7C,I), suggesting that this gene is upregulated significantly in both diseases. The area under the AUC for the signature gene *GPER* in CAD was 0.871 (Figure 7F). In NASH, it was 0.930 (Figure 7L). Once more, both AUC values exceeded 0.7, indicating robust predictive efficacy.

### 3.6 Hypothesis testing of *GPER* across different genders

**FIGURE 8:**
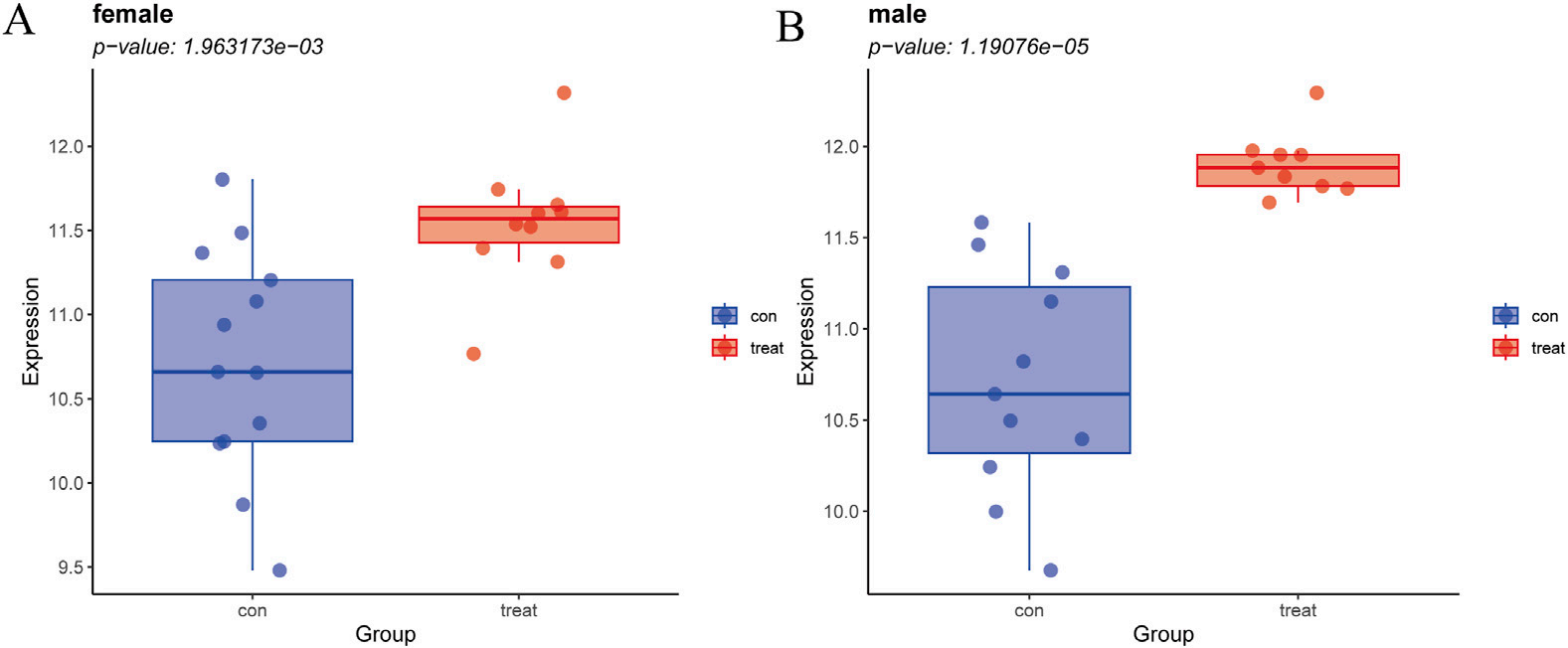
**(A)** Expression levels of GPER in NASH vs. healthy cohorts in females. **(B)** Expression levels of GPER in NASH vs. healthy cohorts in males.

Since *GPER* acts as an estrogen receptor, it functions mainly in women (Morán-Costoya et al., 2021). To avoid the bias caused by gender differences, we verified whether *GPER* still has expression differences in male patients by significance testing. Due to the absence of gender-specific data in the remaining datasets, the NASH dataset GSE89632 was the sole source available for this analysis. The samples were divided into two groups, one comprising male subjects and the other composed of female subjects. We then separately compared the differences between the two groups of patients and normal samples. Male and female patient groups showed statistically significant differences compared to normal subjects (p < 0.001) (Figure 8).

### 3.7 Validating key genes

**FIGURE 9:**
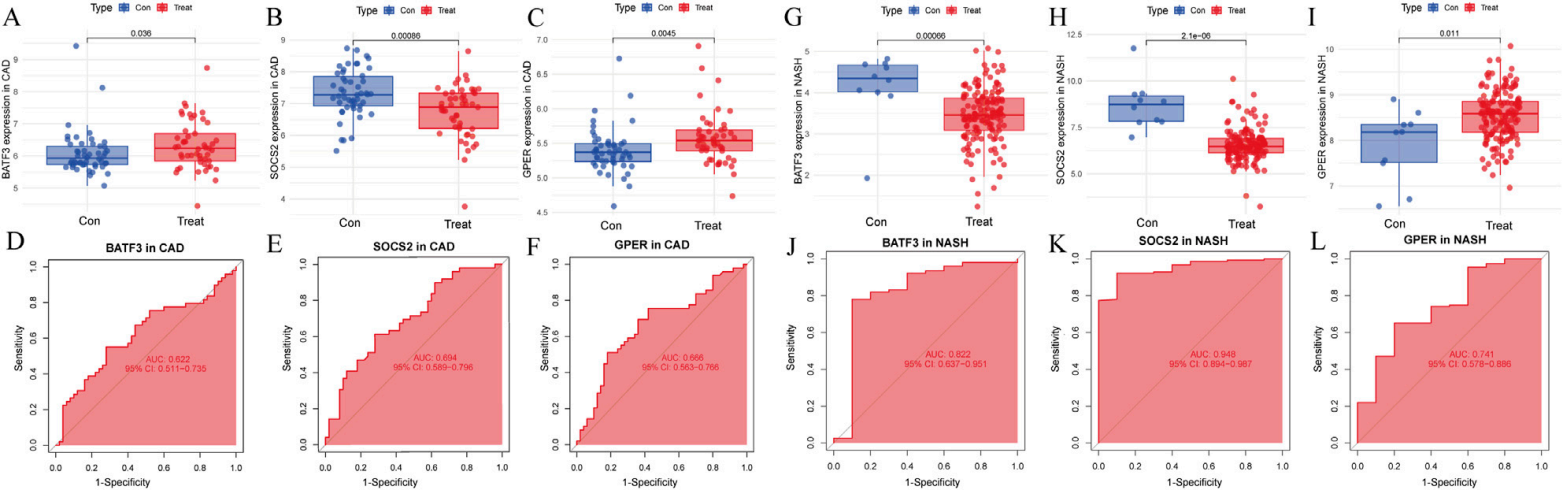
Evaluation of signature genes in GSE66360 and GSE135251. **(A–C)** Expression levels of signature genes in CAD vs healthy cohorts. **(D–F)** ROC curves demonstrating the predictive power of signature genes in CAD. **(G–I)** Expression levels of signature genes in NASH vs healthy cohorts. **(J–L)** ROC curves showcasing the diagnostic efficacy of the signature genes in NASH.

Next, we assessed whether the signature genes were significantly different in diseased and normal tissues in external validation cohorts. We also evaluated the ROC curves of these genes to determine their diagnostic efficacy in predicting NASH and CAD. In the GSE66360(CAD validation cohort), the expression of *GPER* was significantly higher in the disease cohort than in the control cohort. The other genes showed lower expression levels, consistent with the findings in GSE113079 (Figures 9A–C). All three genes in this group were statistically different between the disease and normal groups (p < 0.05). The AUC values derived from the ROC curves for *BATF3*, *SOCS2*, and *GPER* were 0.622, 0.695, and 0.666, respectively (Figures 9D–F). All AUC values were greater than 0.5 but less than 0.7, indicating moderate predictive ability.

In the GSE135251 (NASH validation cohort), *GPER* was once more identified as being highly expressed relative to the control group. In contrast, the other genes displayed lower expression levels, consistent with the findings from GSE89632 (Figures 9G–I). All three genes showed statistically significant differences between the disease and normal groups $\left(p<0.05\right)$ . The AUC values for *BATF3*, *SOCS2* and *GPER* were 0.822, 0.741 and 0.948, respectively. Each AUC value was greater than 0.7, demonstrating robust predictive ability (Figures 9J-K).

### 3.8 GSEA analysis

**FIGURE 10:**
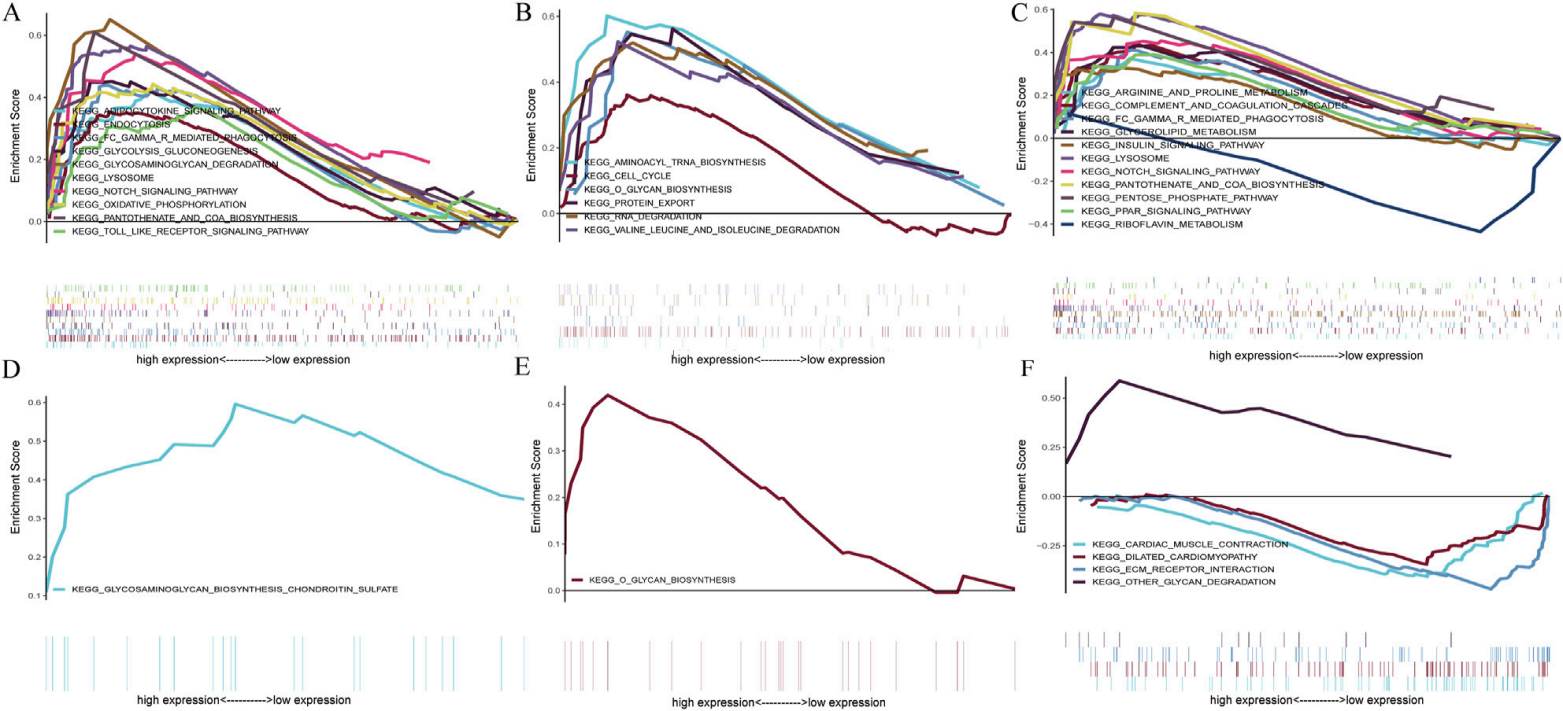
GSEA of the signature genes in CAD and NASH. **(A)** GSEA of *BATF3* in CAD. **(B)** GSEA of *SOCS2* in CAD. **(C)** GSEA of *GPER* in CAD. **(D)** GSEA of *BATF3* in NASH. **(E)** GSEA of *SOCS2* in NASH. **(F)** GSEA of *GPER* in NASH. In each panel, the x-axis represents all genes ranked by log_2_ fold change, and the y-axis shows the running enrichment score.

GSEA analysis assessed the pathways related to the signature genes to identify their relevance to the disease. In the context of CAD, The upregulation of *BATF3* was found to have statistically significant relevance to several pathways, including adipocytokine signaling pathway, endocytosis, Fc gamma R-mediated phagocytosis, glycolysis gluconeogenesis, glycosaminoglycan degradation, lysosome, Notch signaling pathway, oxidative phosphorylation, pantothenate and CoA biosynthesis, and Toll-like receptor signaling pathway (Figure 10A). The upregulation of *SOCS2* was significantly associated with aminoacyl-tRNA biosynthesis, cell cycle, O-glycan biosynthesis, protein export, RNA degradation, and valine leucine and isoleucine degradation (Figure 10B). The upregulation of *GPER* is significantly associated with pathways related to arginine and proline metabolism, glycerolipid metabolism, insulin signaling pathway, etc. Its downregulation was significantly associated with riboflavin metabolism (Figure 10C). In the context of NASH, the upregulation of *BATF3* was significantly associated with glycosaminoglycan biosynthesis of chondroitin sulfate (Figure 10D). The upregulation of *SOCS2* was significantly associated with O-glycan biosynthesis (Figure 10E). The upregulation of *GPER* was significantly associated with other glycan degradation, whereas the downregulation was associated with ECM-receptor interaction, cardiac muscle contraction, and dilated cardiomyopathy (Figure 10F).

In conclusion, *BATF3* is involved in the metabolic pathways related to both diseases. *SOCS2* has been shown to activate the O-glycan biosynthesis pathway in both CAD and NASH. *GPER*’s upregulation is associated with pathways related to glycolipid metabolism in both conditions, while its downregulation is linked to pathways concerning cardiovascular function. This suggests that *GPER* may be important in regulating metabolism and maintaining cardiovascular health.

### 3.9 Immune cell infiltration analysis

**FIGURE 11:**
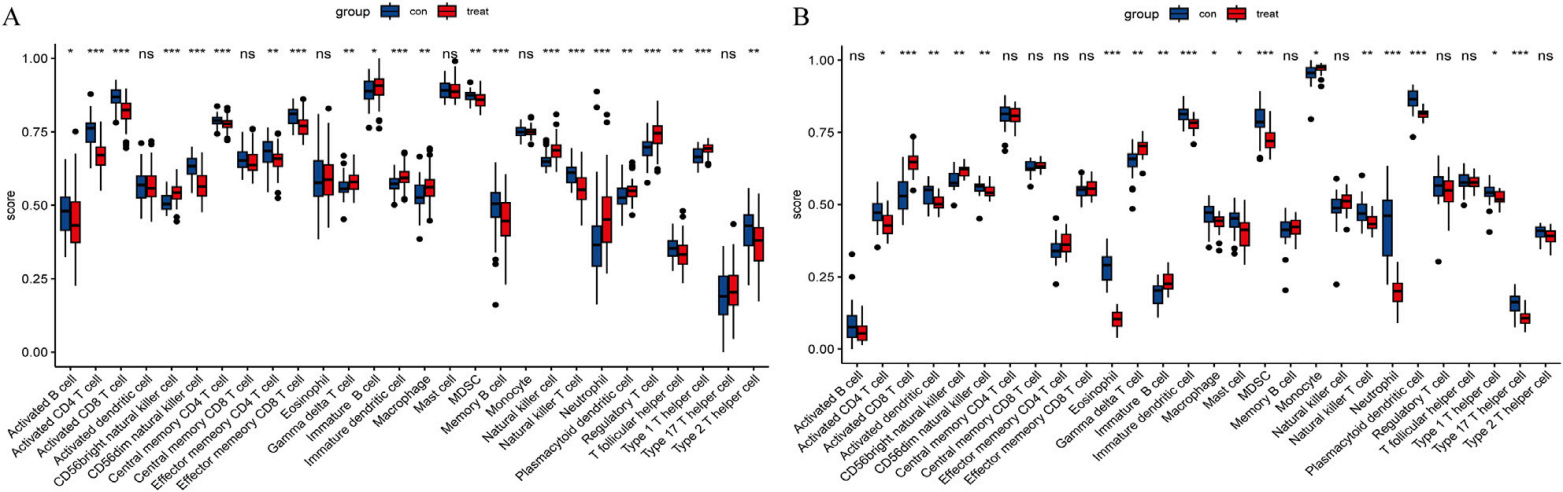
Immune cell infiltration. **(A)** Immune cell infiltration comparison between CAD and healthy cohorts. **(B)** Comparison of immune cell infiltration between NASH and healthy cohorts. “ns” indicates p≥0.05 , * denotes 0.01≤p<0.05 , ** indicates 0.001≤p<0.01 , and indicates p<0.001 . The x-axis represents different immune cells, and the y-axis indicates the ssGSEA score. Each box represents the score distribution of a group: blue boxes represent the healthy group, and red boxes represent the disease group.

For CAD, CAD patients showed higher infiltration levels of CD56bright natural killer cells, gamma delta T cells (γδ T cells), immature B cells, neutrophils, regulatory T cells, etc., compared to the normal group. Conversely, infiltration levels of activated B cells, activated CD4 T cells, activated CD8 T cells, CD56dim natural killer cells, myeloid-derived suppressor cells (MDSC), etc. were lower (Figure 11A). For NASH, the patients showed higher infiltration levels of activated CD8 T cells, CD56bright natural killer cells, γδ T cells, immature B cells, and monocytes compared to the normal group. In contrast, activated CD4 T cells, activated dendritic cells, CD56dim natural killer cells, MDSCs, etc., showed lower infiltration levels (Figure 11B).

Notably, in both diseases, the infiltration levels of CD56bright natural killer cells, γδ T cells, and immature B cells were significantly elevated compared to the normal group, whereas the infiltration levels of activated CD4 T cells, CD56dim natural killer cells, and MDSCs were reduced.

### 3.10 The expression of *BATF3* shows a positive correlation with γδ T cells

**FIGURE 12:**
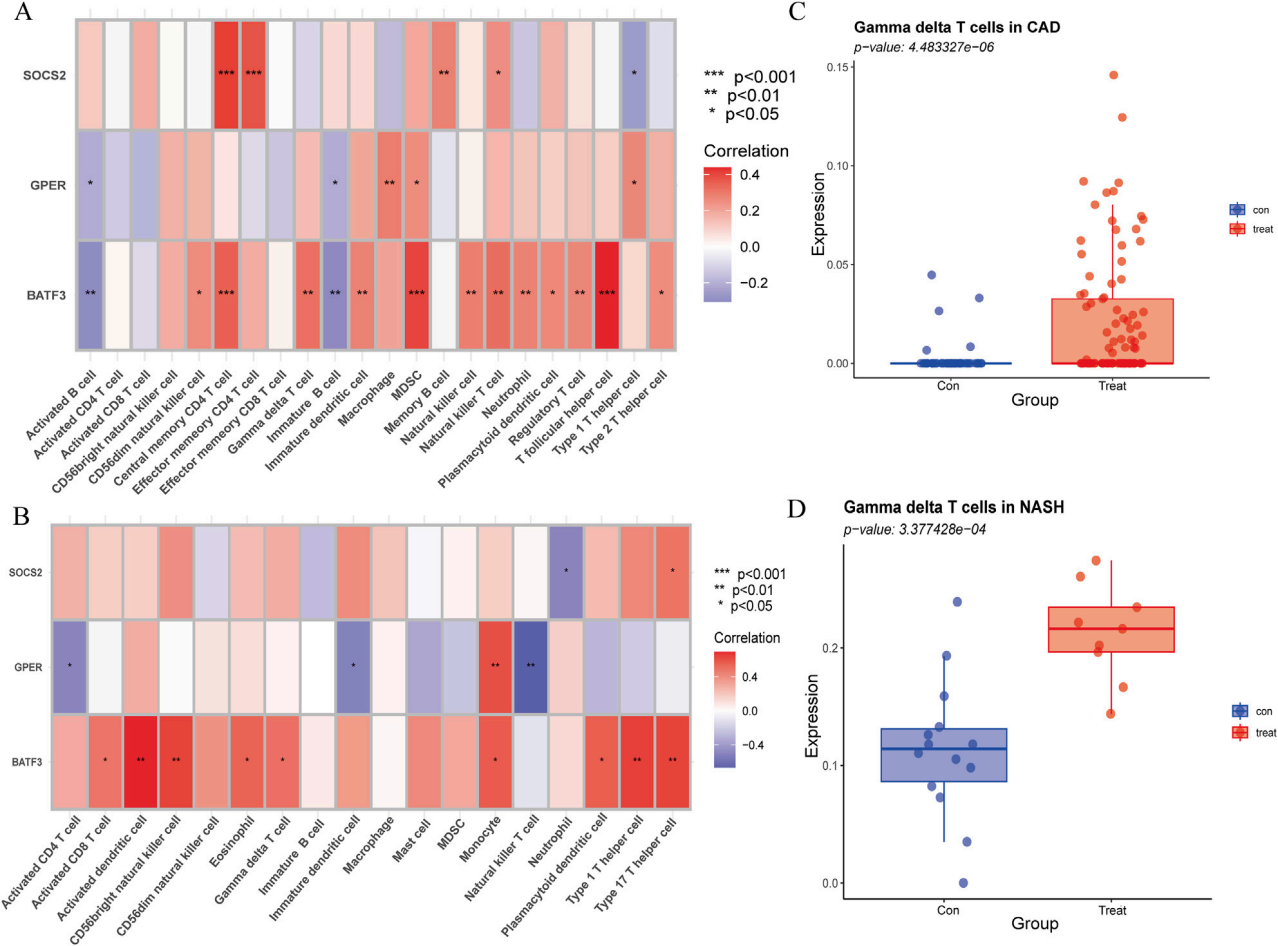
Association of immune cell infiltration with signature genes, validated by CIBERSORT. **(A)** Correlation of signature genes with differences in immune cell infiltration in CAD. **(B)** Correlation of signature genes with differences in immune cell infiltration in NASH. The x-axis represents different immune cell types, and the y-axis represents different genes. The color of each square indicates the corresponding p-value, with the color gradient reflecting the level of statistical significance. **(C)** Gamma delta T cell expression in CAD versus healthy controls using CIBERSORT. **(D)** Gamma delta T cell expression in NASH versus healthy controls using CIBERSORT.

The Spearman rank correlation coefficients between the differential immune cell populations and the feature genes were evaluated (Figures 12A,B). The results of our analysis reveal that there is a positive correlation between γδ T cells and the expression levels of *BATF3* in both disease states examined. As previously stated, the number of γδ T cells is increased in both conditions. To validate the expression trend of γδ T cells in both diseases, we employed the CIBERSORT algorithm. The results obtained from this analysis were consistent with those derived from ssGSEA (Figures 12C,D).

## 4 Discussion

NASH and CAD are common diseases that significantly affect the health of patients. In recent years, increasing epidemiologic, genetic, pathologic, and clinical observational evidence suggests that NAFLD may be an independent risk factor for CAD (Zhang D. et al., 2023). NASH is a subtype of NAFLD with more severe damage and is an important intermediate link in the development from simple fatty degeneration of hepatocytes to liver fibrosis and liver cancer. An epidemiologic study on NASH showed a global prevalence of NASH of more than 15% as of 2016 (Dong et al., 2024). In addition, approximately 20% of NASH patients will develop cirrhosis (Sheka et al., 2020). Moreover, higher risk of CAD in NASH patients than in those with simple steatosis, a Japanese study shows (Niikura et al., 2020). Therefore, further research into the incidence and development mechanism of NASH is especially important. This study is designed to discover the common genetic markers and pathogenesis of NASH and CAD and to evaluate the common immune microenvironment of the two diseases in pursuit of more effective preventive measures, diagnosis, and treatment approaches for these diseases. In this study, WGCNA and differential gene selection were performed on the two diseases to find out the common key gene sets. By performing GO and KEGG functional analysis on 44 potential target genes related to the two diseases, the various biological processes and pathways associated with these genes were uncovered, thereby determining their potential modes of action in the disease. In addition, three machine learning algorithms were applied to identify three signature genes, *BATF3*, *SOCS2*, and *GPER*, and their respective roles in the disease were explored. Finally, ssGSEA was employed to assess the shared immune infiltration in CAD and NASH and to analyze its correlation with signature genes.

At present, the recognized common risk factors for NAFLD and CAD include metabolic syndrome, insulin resistance, dyslipidemia, as well as obesity (Brouwers et al., 2019). While our KEGG pathway analysis confirmed the involvement of shared pathways such as the insulin signaling and FoxO signaling pathways, a deeper understanding of their functional implications is warranted. FoxO transcription factors are downstream mediators of insulin signaling. Their activity is regulated by serine/threonine phosphorylation catalyzed by protein kinase B and other kinases. Prior research has demonstrated their substantial contribution to lipid homeostasis in diet-induced fatty liver disease (Pan et al., 2017), thereby indicating a mechanistic association with NASH pathogenesis. Based on the analysis as mentioned above and conclusions, we postulate that FoxO may influence the pathogenesis of CAD by regulating mechanisms such as insulin signaling, oxidative stress, and inflammatory responses. In light of these findings, the FoxO signaling pathway may serve as a viable intervention target for CAD in conjunction with NASH.

In addition, our study demonstrated that the development of both diseases is also significantly influenced by inflammatory pathways, including cytokine-cytokine receptor interaction, TNF signaling pathway and TGF-beta signaling pathway. Endothelial dysfunction, a hallmark of early-stage CAD, initiates an inflammatory cascade by promoting the infiltration of macrophages and the release of pro-inflammatory mediators, including cytokines and chemokines, thereby amplifying local vascular inflammation (Zouridakis et al., 2004; Ebadi et al., 2022). Studies have demonstrated that levels of inflammatory factors are statistically significantly increased in the epicardial tissue of CAD patients (Zhou et al., 2011). Similarly, NASH is characterized by hepatic lipid accumulation, hepatocyte injury, and fibrosis, all of which are closely linked to an enhanced inflammatory milieu (Thibaut et al., 2022). Notably, TNF-α not only speeds up the progression of NASH but also prevents hepatocyte apoptosis by inducing TNF-α-induced protein 8-like 1. This action helps reduce steatosis and fibrosis, offering protection against NASH (Wu et al., 2021). In conclusion, inflammatory signaling pathways are of great importance in the pathogenesis of both CAD and NASH and may serve as common biomarkers for these diseases.

Basic leucine zipper ATF-like transcription factor 3 (*BATF3*) is a member of the basic leucine zipper transcription factor family and plays a part in controlling gene expression. This process is facilitated by the formation of heterodimers with multiple transcription factors. *BATF3* is a key transcription factor involved in generating CD8α^+^ dendritic cells within lymphoid tissues and CD103^+^ dendritic cells in the skin. Our study shows that *BATF3* levels are decreased in the peripheral blood of patients with CAD and NASH and that this expression is positively correlated with γδ T-cell infiltration in these conditions. Furthermore, elevated levels of γδ T cell expression were observed in both diseases. This finding suggests that *BATF3* might contribute to immune regulation in both diseases. A study on patients with CAD demonstrated elevated γδ T cell expression in atherosclerotic plaque samples, which is consistent with our findings (Liu et al., 2024). However, another study reported a decreasing trend in the proportion of γδ T cells in patients with CAD (Han et al., 2021). This discrepancy may be attributed to differences in sample sources, as the study in question used data from the GSE40231 dataset, which was derived from carotid lesion tissues, whereas our samples were obtained from peripheral blood. Furthermore, a study from China demonstrated that in patients with acute myocardial infarction, certain γδ T-cell subsets exhibited restricted expression, while others were markedly elevated (Chen et al., 2018). This finding suggests that γδ T cells in CAD patients may not be uniformly decreased, but rather exhibit fluctuating expression patterns that are closely associated with specific subsets. Despite the limitations of the GSEA approach employed in our analysis, which precludes the capacity to discern between distinct γδ T-cell subpopulations, our findings align with those of the aforementioned study, collectively suggesting that γδ T cells may have a pivotal function in the development and progression of CAD. Nevertheless, the role of γδ T cells in NAFLD remains underexplored. Our study demonstrates that γδ T cells are upregulated in NASH. γδ T cells can detect pathogens and stimulate dendritic cell maturation, functional activation, migration, and antigen presentation. There has been evidence that mature dendritic cells exhibit elevated levels of costimulation molecules in the context of CD1-restricted T cells and reduced antigen uptake when there are CD1-restricted T cells around (Leslie et al., 2002). It is postulated that the downregulation of *BATF3* may impair dendritic cells function, thereby affecting γδ T cells and resulting in aberrant γδ T cell infiltration in NASH and CAD. The precise mechanisms remain unclear and require further investigation.

Suppressor of Cytokine Signaling 2 (*SOCS2*) is a critical protein that plays a significant role in modulating cytokine responses. It acts as a classic negative regulator of the JAK/STAT signaling pathway (Cabrera-Galván et al., 2023). *SOCS2* can mitigate inflammation by attenuating inflammasome signaling pathways, including inhibiting the NF-κB pathway to decrease macrophage apoptosis. This action helps reduce inflammation and slows the progression of NASH (Xiao et al., 2023). The observed reduction of *SOCS2* in liver tissue under high-fat diet conditions and its inverse correlation with NASH severity support its protective role in liver homeostasis (Li S et al., 2021; Zhang Z. et al., 2023). It is consistent with our results. Research suggests that *SOCS2* may interact with the JAK/STAT3 pathway to cause coronary CAD in diabetic patients (Sheng et al., 2017). While the JAK/STAT3 pathway typically exerts cardioprotective effects by inhibiting apoptosis, preserving mitochondrial integrity, and promoting angiogenesis, its overactivation may trigger proinflammatory cascades under certain conditions (Yasukawa et al., 2012). For instance, in ApoE^−/−^ mice, JAK2/STAT2 activation has been shown to exacerbate macrophage-mediated inflammation in response to homocysteine (Xu et al., 2022). Therefore, we speculate that downregulation of *SOCS2* may reduce its inhibitory effect on the JAK2/STAT2 pathway, leading to an enhanced macrophage inflammatory response in CAD.

Our GSEA analysis of *SOCS2* demonstrated that *SOCS2* expression is associated with O-glycosylation biosynthesis in both diseases. Glycosylation is among the most common post-translational modifications in eukaryotic cells and is considered a key factor in the progression of NAFLD (Zhan et al., 2016). The glycosylation of apolipoprotein B, extensive N-linked glycosylation of fatty acid translocase, and the interaction between carbohydrate response element-binding protein and O-GlcNAc transferase have all been closely associated with metabolic dysregulation and inflammatory responses in the progression of NAFLD (Ihara et al., 1998; Guinez et al., 2011). Additionally, previous studies have shown that patients with NASH exhibit a distinct glycosylation pattern characterized by an increase in core-fucosylated biantennary glycans and a decrease in galactosylated, non-fucosylated biantennary glycans, which can help distinguish between NAFLD and NASH(Verhelst et al., 2020). In CAD, leukocyte recruitment is a critical step in the formation of arterial plaques, and the function of adhesion molecules and chemokine receptors involved in leukocyte recruitment is determined by proper post-translational glycosylatio (Lowe, 2002; Frommhold et al., 2008). For example, the inhibition of α2,3-sialyltransferase IV, α2,3-fucosyltransferase, and core 2 β1,6-galactosyltransferase I has been shown to suppress the development of atherosclerosis (Pu and Yu, 2014). Our study suggests that SOCS2 may influence the progression of both diseases through the O-glycosylation biosynthesis pathway and serve as a potential molecular link between them. However, this remains a hypothesis based solely on bioinformatics predictions and requires further validation through *in vivo* and *in vitro* experiments to determine whether and how SOCS2 is involved in the regulation of this pathway.

G-protein coupled estrogen receptor 1 (*GPER*) is a seven-transmembrane domain receptor that interacts with estrogen. It acts mainly by activating several downstream signaling pathways. (Revankar et al., 2005; Prossnitz and Barton, 2011). Emerging evidence suggests that *GPER* plays a critical role in the progression of NASH by activating the AMPK signaling pathway. This activation modulates lipid metabolism by promoting lipogenesis while simultaneously inhibiting lipolysis in both male and female mice. The net effect is a reduction in hepatic fat accumulation and the downregulation of fibrosis-related genes, ultimately alleviating liver fibrosis (Li et al., 2024). *GPER* is also associated with immune cell infiltration, fibrosis, and the release of inflammatory factors in the liver. Additionally, it is correlated with HDL and LDL levels in the blood. In endothelial cells, the activation of *GPER* by estrogen decreases the transcytosis of LDL cholesterol into these cells, thereby providing indirect protection to the vasculature (Ghaffari et al., 2018). *GPER* activation promotes relaxation of the coronary arteries and reduces smooth muscle cell proliferation and migration. This helps prevent or reverse the progression of coronary atherosclerotic disease by increasing coronary blood flow in the affected arteries (Barton and Prossnitz, 2015).

However, the incidence of both NASH and CAD is sexually dimorphic. The incidence and severity of CVD and NAFLD are lower in premenopausal women than in men of the same age and postmenopausal women (Mouat et al., 2018; Lonardo et al., 2019). Higher levels of oestrogen may explain the lower prevalence in women. And *GPER* is the primary receptor that mediates estrogen levels (Li et al., 2024). However, studies report no significant sex-based differences in *GPER* expression across major tissues such as the aorta, heart, and kidney in *Sprague-Dawley* rats, suggesting estrogen may exert similar signaling efficiency in both sexes (Hutson et al., 2019). In addition, male *GPER*-deficient mice exhibit an overall increase in body fat, insulin resistance and a pro-inflammatory phenotype (Sharma et al., 2013), indicating that *GPER* may also play a crucial protective role in males. These suggest that *GPER* also plays a protective role in males. Our study indicates that *GPER* expression is significantly associated with the development of NASH in both men and women, reinforcing its importance in men as well. Thus, *GPER* may serve not only as a sex-independent signature gene but also as a potential therapeutic target. Future research is needed to clarify the mechanistic roles *GPER* plays specifically in males and how its modulation might be leveraged therapeutically.

We need to acknowledge the limitations of our study. First, the data were obtained from a public database with a relatively limited sample size, which may have some impact on the performance of the machine learning model. The small sample size may lead to an unstable model training process and increase the risk of overfitting. In addition, the small sample size prevents ensemble methods such as RF from fully exploiting their benefits. In addition, the data in public databases may have certain biases, such as selection bias and population bias. The CAD and NASH populations in this study were from China and Canada, respectively. However, we obtained good validation results in external datasets from the United States and the United Kingdom. Nonetheless, the study remains a bioinformatics-based investigation without supporting *in vivo* or *in vitro* experimental validation. Therefore, further molecular biology experiments and clinical studies are strongly recommended to validate the biological functions and clinical significance of the signature genes identified in this study.

## 5 Conclusion

This study identified three signature genes, *BATF3*, *SOCS2*, and *GPER*, shared at the transcriptional level in both NASH and CAD. Additionally, we demonstrated that insulin resistance-related and inflammation-related pathways represent common mechanisms underlying these two diseases. Moreover, we examined the infiltration of immune cells in CAD and NASH and their relationship with the characteristic genes, offering new perspectives on the role of immunity in CAD complicated by NASH. Nevertheless, further research is needed to help us understand the mechanisms underlying the common pathways and to address the potential limitations of this study.

## Data Availability

Publicly available datasets were analyzed in this study. This data can be found here: GSE113079: https://www.ncbi.nlm.nih.gov/geo/query/acc.cgi?acc=GSE113079 GSE66360: https://www.ncbi.nlm.nih.gov/geo/query/acc.cgi?acc=GSE66360 GSE89632: https://www.ncbi.nlm.nih.gov/geo/query/acc.cgi?acc=;GSE89632 GSE135251: https://www.ncbi.nlm.nih.gov/geo/query/acc.cgi?acc=GSE135251 GEO database: https://www.ncbi.nlm.nih.gov/geo/.

## Abbreviations

CAD, Coronary artery disease; NASH, Non-alcoholic steatohepatitis; WGCNA, Weighted gene co-expression network Analysis; GO, Gene ontology; KEGG, Kyoto encyclopedia of genes and genomes; GSEA, Gene set enrichment analysis; ssGSEA, Single-sample gene set enrichment analysis; γδ T Cells, gamma delta T cells.
