# Supplementary material for: Identification of biomarkers between coronary artery disease and non-alcoholic steatohepatitis: a combination of bioinformatics and machine learning
Source: Front Genet. 2025 Jul 17;16:1573621. doi: 10.3389/fgene.2025.1573621 (PMC12310482; doi:10.3389/fgene.2025.1573621)
Supplement: Supplementary file 1 [file DataSheet1.pdf]

Supporting Table. List of genes selected based on least absolute shrinkage and selection operator (LASSO)

LASSO genes			
BATF3	CEBPA	DHRS13	ERN1
FOXC1	FOXO1	GPD1	GPER
SEC14L2	SLC16A13	SOCS2	TGFB3
